# Implementation and Utility of the Da Vinci SP (Single Port) in Pediatric Urology

**DOI:** 10.1007/s11934-024-01231-7

**Published:** 2024-10-03

**Authors:** Lucas A. Arney, Randall G. Bissette, Jordan M. Smith, Christopher E. Bayne

**Affiliations:** 1 Virginia Tech Carilion School of Medicine, Roanoke, VA USA; 2 Department of Urology, University of Florida College of Medicine, Gainesville, FL USA; 3 Section of Urology, Department of Surgery, Virginia Tech Carilion School of Medicine, Carilion Clinic, Roanoke, VA USA; 4 Pediatric Urology, 4348 Electric Rd, 24018 VA Roanoke, USA

**Keywords:** Pediatric urology, Robotic surgery, Single port, Single-port, SP

## Abstract

**Purpose of Review:**

Detail the evolution, utility, and future directions of the da Vinci SP® in pediatric urology, focusing on perioperative outcomes and intraoperative details.

**Recent Findings:**

The SP has been safely and successfully utilized in various pediatric urological procedures, from pyeloplasty to nephroureterectomy to appendicovesicostomy. Reports indicate mixed operative times but similar hospital stays and postoperative outcomes compared to multiport (MP) robotic surgery. The learning curve for transitioning from MP to SP systems in pediatric patients appears manageable, though the smaller abdominal circumference in children poses a notable challenge. This review assumes that SP systems will primarily be acquired for adult services, not considering initial and ongoing costs to hospital systems.

**Summary:**

The SP serves as a complementary option, rather than a replacement, for MP robotic surgery in pediatric urology, offering potential advantages in specific scenarios. Cosmetic outcomes with the SP appear at least as favorable as MP surgery, but further research is needed. Future research should focus on patient-centered outcomes to optimize SP robotic surgery use in pediatric patients.

## Introduction

The da Vinci SP^®^ (Intuitive Surgical Inc., Sunnyvale, California) is the novel fourth-generation model of the da Vinci robotic surgical platforms. The SP is designed to perform complex procedures through a single 2.5 cm canula. This canula contains four instrument lumens, permitting three 6 mm EndoWrist^®^ instruments with a wrist and elbow joint and a 8 mm three-dimensional, high-definition endoscope. The endoscope articulates from 0 to 30 degrees from either a top or bottom position, improving visualization in challenging-to-reach areas.

It must be stated from the onset that use of the SP in children remains controversal. Some pediatric urologists have been outspoken against use of the system in children. One common criticism has been that the U.S. Food and Drug Administration (FDA) has not approved the SP for pediatric surgical indications. Importantly, the FDA has not approved the da Vinci Xi^®^ for pediatric surgery indications, either. The da Vinci Si^®^ held pediatric indications for pyeloplasty and ureteral reimplantation; however, this system is being phased out of production. As such, all pediatric robotic surgery, whether performed with the SP or the Xi, is presently considered “off-label”.

 It should be acknowledged that single port (SP) and single incision minimially-invasive surgery is not new to pediatric urology. Laparoendoscopic single site (LESS) surgery has been published from both periumbilical incisions [1] and low transverse incisions [2]. Surgeons pioneering these approaches have highlighted ergonomic challenges of operating in confined pediatric spaces with cross-handed instrumentation, especially without the use of articulating instruments. Recently, Liu and colleagues in Wuhan, China reported using the da Vinci Xi to perform an infant pyeloplasty via a single periumbilical incision with a laparoscopic gel port [3].

A key to understanding use of the SP is that at least 10 cm of working distance is necessary between the tip of the robotic canula and the target anatomy. This distance ensures the instruments have enough room to exit the canula, flex at the elbow, and triangulate at the target anatomy. If there is less than 10 cm working distance between the trocar tip and target anatomy, surgeons will not be able to fully articulate the instruments, instrument clashes will increase, and the surgeon will find instrument movements to be jerky and unsafe. As working space within the pediatric abdomen and pelvis can be limited, some authors have adapted a “floating dock” approach. The floating dock concept describes a technique in which the robotic canula is directed through the gel cap of a wound access sleeve already placed within the body. The robotic canula “floats” outside the body but within the access sleeve, deploying the robotic instruments outside the body and effectively increasing and optimizing distance from canula tip to target anatomy. The floating dock also preserves sterility and maintains insufflation pressure.

In this review, we provide an update on published reports of use of the SP in pediatric urology. The role for SP robotic surgery in pediatric urology is evolving. Some have reported longer operative times and difficulty in adapting the technology to pediatric patients compared to traditional multiport (MP) robotic surgery. Though reported techniques and experiences remain sparce, there are attributes that make SP robotic surgery inherently attractive in children: improved instrumentation compared to laparoscopy, cosmetically attractive incision compared to conventional MP robotic surgery, and the ability to perform upper tract and lower tract reconstructive, expirtation, and specimen extraction through a single, 3 cm incision.

## Reported SP Experiences in Pediatric Urology

In 2021, Granberg and colleagues at Mayo Clinic published the first use of the SP in pediatric urology [4]. The report primarily detailed a pyeloplasty in a 10-year-old girl. The authors cited the SP had been utilized in 6 additional pediatric patients from 23 months to 14 years. The authors utilized a single incision for all cases and directly inserted the SP canula through the abdominal wall and into the peritoneal cavity. With the exception of mean operative time reported at 120 min, operative details were largely omitted. The group republished these 7 cases in a video report featuring an SP robotic appendicovesicostomy in a 14 year-old patient. This case was particularly novel in that it utilized a previous gastrostomy scar for placement of the SP cannula [5]. The authors reported the same cohort of patients in a third publication in 2023 [6]. Between the 3 publications of the 7 patient cohort, the authors noted the necessary 10 cm working distance and loss of insufflation with introduction of assistant laparoscopic instruments through the instrument sleeve lumens as limitations to the SP in children. Notably, the authors directly inserted the robotic trocar into the abdomen and did not utilize the floating dock concept published in subsequent experiences. The authors reported a “minimal learning curve” in utilizing the SP, different from observations noted in subsequent publications from other groups [7, 8].

Kang et al. published the world’s first SP case series in pediatric urology in July 2021 [9]. The South Korean series compared surgical outcomes between SP robot-assisted laparoscopic (RAL) pyeloplasty (S-RALP) versus conventional MP robot-assisted laparoscopic pyeloplasty (M-RALP). The authors compared 15 S-RALP patients to 31 M-RALP patients. For the S-RALP group, the authors utilized a periumbilical approach with a gel access sleeve to achieve a floating dock. Median operative time was shorter for S-RALP at 2.4 h compared to 3.0 h for M-RALP. Console times were also reduced (1.5 h for S-RALP versus 2.2 h for M-RALP). Conversion to open surgery, analgesic use, estimated blood loss (EBL), postoperative pain scores, postoperative complications, and hospital stay duration were comparable between groups.

Smith and colleagues at University of Florida (UF) published their experience with the SP in August 2023 [7]. Unique to this experience was the reported evolution of surgical technique: initial cases were performed with a low transverse floating dock approach and a separate periumbilical assistant trocar. Middle cases were performed with a periumbilical floating dock that included an assistant port. Later cases were performed with a 3 cm incision and floating dock directly over the pubic tubercle. The authors compared outcomes from their initial 11 S-RALP cases to 5 M-RALP cases during the same timeframe. The 11 patients who underwent S-RALP were older patients, ranging from 8 months to 17 years, while the 5 who underwent M-RALP ranged from 3 months to 14 months. S-RALP procedures had a longer overall operative time than M-RALP, with S-RALP taking a median 384 min versus 299 min for M-RALP. There was improvement in the latter five S-RALP cases. In response to criticism of operative times, the authors pointed out their reported operative times acounted for the learning curve of a new technology, the evolution in technique, interrupted anastomoses, and included all operative time, from initiation of cystoscopy to repositioning, incision and robotic docking, and completion of all skin closure(s). All other outcomes, including hospital stay, opiod administration, and surgical success, were comparable between groups.

The most recent adaptation of the SP in pediatric urology comes from the Cleveland Clinic where Chavali and colleagues reported their extraperitoneal pyeloplasty technique applied to 6 patients, ranging from 12 months to 16 years [8]. The authors utilized an off-midline, low transverse approach to access the ipsilateral retroperitoneum and affected kidney. Total operative time ranged from 178 to 240 min. Median hospital stay was 1 day, with 2 of the patients discharged on the same day as surgery. The authors reported absence of conversion to open, readmissions, or complications, and reported surgical success in all 6 cases. The team acknowledged a potentially steep learning curve to using the SP in the retroperitoneal space of children.

## Unique Considerations and Applications where the SP may add Value

There are particular considerations in which an SP approach may provide unique value to children undergoing robotic surgery.

One area of value may be cosmetic outcome of the surgical scar. An SP transperitoneal approach to the kidney involves a 2.7–3 cm incision directly over the pubic tubercle, hidden well beneath virtually all bathing suits and undergarmets. Using a floating dock and positioning considerations, the renal anatomy is readily accessible [10]. Preliminary data on patient experience and scar perception following pediatric SP transperitoneal pyeloplasty compared to open and MP pyeloplasty has been promising [11]. A pilot study at UF compared validated patient-centered outcome surveys of 16 families whose children underwent SP surgery to those whose children underwent open and MP pyeloplasty during the same time span. For children 10 years and older, patients were surveyed, as well. Data suggest families and patients view the SP experience similarly to those who undergo MP surgery. In the domains of scar perception, families and patients view their SP scars at least as favorably, if not more favorably, than the open and MP patients. More patient-centered data are needed to draw firm conclusions.

Beyond cosmetic considerations, an SP approach to pediatric urological robotic surgery may offer technical advantages in unique clinical scenarios. To date, these unique scenarios have included (1) utilizing previous open incisions and minimizing scar footprint, (2) using a single low, hidden transverse incision for combined nephrectomy and specimen extraction, (3) combining upper tract renal or ureteral surgery with open lower tract surgery, all performed through through the same low, hidden transverse incision, (4) accessing the renal retroperitoneal space via an offset transverse incision beneath the anterior superior iliac spine, and (5) accessing the deep pelvis via a transvesical approach for complex reconstruction demands.

As Parikh and colleagues have shown, an SP approach may be particularly useful in the setting of previous open incisions [5]. These authors utilized a previous gastrostomy closure incision for SP appendicovesicostomy creation. The authors were able to complete the procedure without making any additional incisions for assistant trocars.

At UF, surgeons have previously described using a single, low transverse incision for combined robotic upper tract and open lower tract urinary reconstruction and extirpative extraction [12, 13]. When a nephrectomy is necessary in combination with distal ureteral or bladder reconstruction, a single incision makes intuitive sense given the need for a specimen extraction site. Though the authors only performed the upper tract surgery in a transperitoneal approach, one could imagine a retroperitoneal approach using a low transverse single-incision if both upper and lower tract surgery is needed (e.g., proximal ureteroureterostomy and distal ureterectomy in the case of complex complete ureteral duplication surgery).

A final demonstrated application of an SP approach is the ability to work within the deep pelvis for transvesical applications. The Cleveland Clinic group has reported the SP to be uniquely useful for a transvesical approach to a vesicovaginal fistula repair in a 9-year-old female with extensive abdominal surgical history and limited transvaginal access [14].

## Limitations

The anatomic and technical challenges to using the SP in children center on the 2.5 cm trocar width, the necessary ≥ 10 cm working distance between trocar tip and target anatomy, and the limited working space within the pediatric patient. As noted earlier, the floating dock helps increase working distance and feasibility for SP surgery in children. Perhaps counter-intuitively, locating the robotic trocar outside the body has introduced other working space difficulties: flattening the angle of the robotic boom in its approach to the target anatomy, which may on occasion create positional clashing between the robotic boom and instrument drives and the patient. When docking the SP from a low, transverse incision directly over the pubic tubercle, special positioning precautions can help minimize clashing between instrument drives and the lower extremities [10].

The most notable limitation to widespread use of the SP in pediatric urologist is the availability of the platform. It is unlikely that free-standing children’s hospitals can justify the cost of the SP given the versitility of the Xi. Until later generations of the SP can accommodate the unique anatomic challenges to operating in young children, the only pediatric urologists that will likely have access to the SP will be those associated with primarily adult health systems.

## Future Directions

Given the increased operative time and steep learning curve demonstrated by some early adopters of the SP, continued use of the SP in pediatric urology should be performed alongside further research investigations into two specific domains. The first domain is patient-centered outcomes research to investigate whether the single incision provides patients an advantage regarding pain, perceived surgical experience, and scar perception over the conventional multiport robotic approach. Future research in this area ought to compare the single SP incision to a multiport hidden incision endoscopic (HIdES) approach [15] as it is expected patients would be more satisfied with HIdES incisions than conventional transperitoneal robotic trocars.

The second research domain of interest to demonstrate value of SP in pediatric urology is surgical complications and length of hospital stay. A panacea in pediatric urology would be same-day discharge, without sacrificing surgical success or complication rates, for complex upper tract reconstruction, all performed through a single, concealed incision. Retroperitoneal reconstruction may represent an opportunity to achieve this triad. To date, the Cleveland Clinic group is the only to show this these surgeries with same-day discharge. The next reasonable step would be to lower the incision until it is well beneath the anterior superior iliac spine and bathing suit line.

## Conclusions

The SP has been used safely and effectively in pediatric urology at four institutions: Mayo Clinic, Yonsei University in South Korea, University of Florida, and Cleveland Clinic. The technology has not been utilized uniformly across institutions. As such, the experiences reported in broad series are not directly comparable. The harked benefit of the SP robot has been a singular incision. The potentially improved cosmetic outcome and post-operative pain scores have only been preliminarily investigated at one center, and patient volumes were small [11]. Operative times may be longer, and some have reported a steep learning curve, in transitioning from multiport to SP surgery. Unique applications for the SP include single incision upper and lower tract surgery (e.g., nephroureterectomy or combined robotic upper tract and open lower tract surgery) and same-day retroperitoneal surgery. These unique applications, in combination with potential for improved scar perception in the context of equivalent operative outcomes, justifies continued use and investigation in appropriate settings.

## Key References

- Parikh N, Boswell T, Findlay B, Gargollo P, Granberg C. Single-port robotic pyeloplasty in a pediatric patient. Videourology 2021:53. 10.1089/vid.2020.011.
  - First published use of SP in pediatric urology from the Mayo Clinic.
- Granberg C, Parikh N, Gargollo P. And then there was one … incision. First single-port pediatric robotic case series. J Pediatr Urol. 2023;19(4):426.e1-.e4. 10.1016/j.jpurol.2023.03.038.
  - Case series from the Mayo Clinic that included the earliest published use of SP in pediatric urology.
- Smith JM, Hernandez AD, DeMarco RT, Bayne CE. Early Experience With Pediatric Single-port Robotic Pyeloplasty Compared to Multiport Robotic Cohorts. J Urol. 2023;210(2):236-8. 10.1097/ju.0000000000003551.
  - First North American case-control series from University of Florida detailing evolution of surgical technique up to the time of publication and comparing SP and MP experience during the timeframe
- Chavali JS, Frainey B, Ramos R, et al. Single-port robotic extraperitoneal pediatric pyeloplasty using low anterior access: Description of technique and initial experience. J Pediatr Urol. 2024. 10.1016/j.jpurol.2024.01.009.
  - Latest series on SP use in pediatric urology from the Cleveland Clinic detailing entirely extraperitoneal approach.
- Kang SK, Jang WS, Kim SH, Kim SW, Han SW, Lee YS. Comparison of intraoperative and short-term postoperative outcomes between robot-assisted laparoscopic multi-port pyeloplasty using the da Vinci Si system and single-port pyeloplasty using the da Vinci SP system in children. Investig Clin Urol. 2021;62(5):592-9. 10.4111/icu.20200569.
  - World’s first SP case-control series in pediatric urology, comparing SP to MP experience, from Yonsei University in South Korea.

## Data Availability

No datasets were generated or analysed during the current study.
